# A Fatal Case of Spondylodiscitis Complicated by Aortic Fistulization and Mycotic Pseudoaneurysm Formation

**DOI:** 10.7759/cureus.111544

**Published:** 2026-06-26

**Authors:** Zakaria Toufga, Aymane El Farouki, Mohamed Abdellaoui, Rachida Saouab, Hassan En Nouali

**Affiliations:** 1 Department of Radiology, Military Hospital Mohammed V, University Hospital of Ibn Sina, Rabat, MAR; 2 Department of Radiology, Military Hospital Mohammed V, Mohammed V University, Rabat, MAR; 3 Imaging Department, Military Hospital Mohammed V, Mohammed V University, Rabat, MAR

**Keywords:** aortic fistula, mycotic pseudoaneurysm, septic shock, spinal infection, spondylodiscitis, staphylococcus aureus, vertebral osteomyelitis

## Abstract

Spondylodiscitis, a pyogenic infection of the intervertebral disc and adjacent vertebral bodies, is an uncommon but serious condition that may progress to life-threatening vascular complications when the infective process erodes into adjacent great vessels. We report the case of a 60-year-old woman who presented with a three-week history of severe, progressive low back pain, high-grade fever, and chills. Laboratory workup revealed marked leukocytosis and a markedly elevated C-reactive protein level, and blood cultures yielded *Staphylococcus aureus (S. aureus)*. MRI of the thoracolumbar spine demonstrated T12-L1 infectious spondylodiscitis with an anteriorly extending destructive inflammatory tract that was shown on MRI to form a direct fistula into the abdominal aorta, associated with a large mycotic pseudoaneurysm. Emergency aortic repair was planned; however, the patient deteriorated rapidly and developed refractory septic shock, dying within hours of diagnosis. This report underscores the catastrophic potential of vertebral osteomyelitis when complicated by aortic fistulization and mycotic pseudoaneurysm formation, highlights the diagnostic value of contrast-enhanced MRI, and emphasizes the need for early multidisciplinary intervention for such critically ill patients.

## Introduction

Infectious spondylodiscitis is defined as a pyogenic or granulomatous infection involving the intervertebral disc space and the adjacent vertebral end plates. Although its incidence has been increasing in recent decades, largely due to the rising prevalence of immunocompromising conditions, intravenous drug use, and invasive spinal procedures, it remains a relatively uncommon diagnosis, with an estimated annual incidence of 2.4 per 100,000 population in developed countries [1,2]. *Staphylococcus aureus (S. aureus)* is the most frequently isolated causative organism, accounting for approximately 30-50% of pyogenic cases [3].

The clinical course of spondylodiscitis is generally insidious, and delayed diagnosis is common. In most cases, antimicrobial therapy, with or without surgical debridement, results in a satisfactory outcome. However, a subset of patients develops severe complications, including epidural abscess, vertebral collapse with neurological compromise, and, much less commonly, extension of the infectious process into adjacent vascular structures [4]. Because the anterior vertebral bodies and prevertebral tissues lie in close proximity to the abdominal aorta, advanced anterior extension of infection may, in rare cases, involve the aortic wall, resulting in infectious aortitis, pseudoaneurysm, or fistula. Aortic involvement in the form of a vertebro-aortic fistula or a contiguous mycotic aneurysm/pseudoaneurysm represents one of the rarest and most lethal complications of spinal infection, with a reported mortality rate ranging from 50% to 80% despite aggressive surgical or endovascular repair [5,6].

We report a fatal case of thoracolumbar spondylodiscitis due to *S. aureus* that was complicated by direct fistulization into the abdominal aorta with formation of a large mycotic pseudoaneurysm. We discuss the pathophysiological mechanisms, imaging findings, and therapeutic considerations pertinent to this devastating complication.

## Case presentation

A 60-year-old woman with no previously documented medical history presented to the emergency department with a three-week history of severe, progressive low back pain localized to the thoracolumbar region, accompanied by high-grade fever and rigors. She denied any preceding trauma, recent invasive procedure, or history of intravenous drug use. She reported no weight loss, night sweats, or urinary symptoms. She was a non-smoker and denied alcohol use. On physical examination, the patient appeared acutely ill and febrile (temperature 39.4 °C). Blood pressure was 98/62 mmHg, heart rate was 118 beats per minute, respiratory rate was 22 breaths per minute, and oxygen saturation was 94% on room air. Musculoskeletal examination revealed marked percussion tenderness over the thoracolumbar junction (D12-L1 level) with significant paraspinal muscle spasm. Neurological examination of the lower extremities was intact. Abdominal examination demonstrated mild diffuse tenderness without guarding, rigidity, or palpable pulsatile mass.

Laboratory investigations showed a white blood cell count of 16,500 cells/mm^3 ^(reference range: 4,000-11,000 cells/mm^3^) with a neutrophilic predominance of 89%, a C-reactive protein level of 289 mg/L (reference range: <5 mg/L), an erythrocyte sedimentation rate of 112 mm per hour, and a serum procalcitonin level of 18.4 ng/mL. Renal and hepatic function tests were within normal limits. Blood cultures drawn at the time of admission - two sets from separate peripheral venipuncture sites - turned positive within 11 hours of incubation for *S. aureus* in all four bottles. Due to the patient’s rapid clinical deterioration, repeat blood cultures were not obtained, and the definitive antibiotic susceptibility testing results became available only post-mortem. This antibiogram confirmed a methicillin-sensitive *S. aureus* (MSSA) phenotype, showing susceptibility to oxacillin, cefazolin, levofloxacin, and clindamycin, but resistance to penicillin G. Regarding the origin of the infection, a thorough clinical history and physical examination revealed no clear primary source for the MSSA bacteremia, such as skin or soft tissue infections, intravascular devices, or recent surgical wounds.

MRI of the thoracolumbar spine was performed on a 1.5-Tesla unit using a standard spinal protocol. T2-weighted sequences demonstrated confluent hyperintensity involving the D12-L1 intervertebral disk and the adjacent vertebral end plates (Figure 1), indicative of disk-space infection with associated vertebral osteomyelitis. Post-gadolinium T1-weighted sequences (Figures 2-3) showed intense heterogeneous enhancement of the affected disk and vertebral bodies, confirming the inflammatory/infectious nature of the lesion. Critically, the post-contrast sequences also delineated an anteriorly directed destructive inflammatory tract extending from the infected disk space through the anterior longitudinal ligament and the prevertebral soft tissues, establishing a direct communication - a fistulous tract - with the anterior wall of the infrarenal abdominal aorta. A large, irregularly shaped saccular outpouching was identified at the site of aortic wall involvement, measuring approximately 7.7 x 5 cm in maximum axial diameter, with heterogeneous T1 signal and peripheral enhancement consistent with a mycotic pseudoaneurysm. No epidural abscess or spinal cord compression was identified.

**Figure 1:**
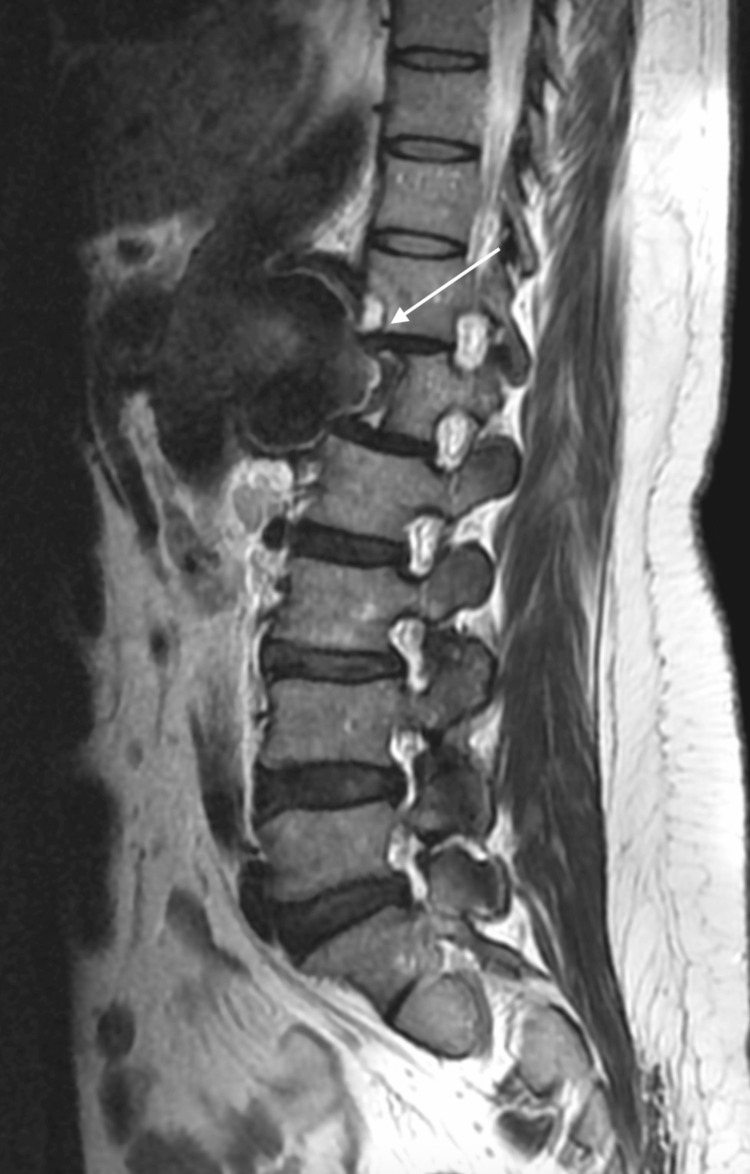
Sagittal T2-weighted MRI of the thoracolumbar spine The image demonstrates confluent hyperintensity involving the D12-L1 intervertebral disk and the adjacent vertebral end plates, indicative of disk-space infection with associated vertebral osteomyelitis. A significant anteriorly extending destructive inflammatory tract has established a direct fistulous communication with the abdominal aorta, forming a large mycotic pseudoaneurysm (white arrow) MRI: magnetic resonance imaging

**Figure 2:**
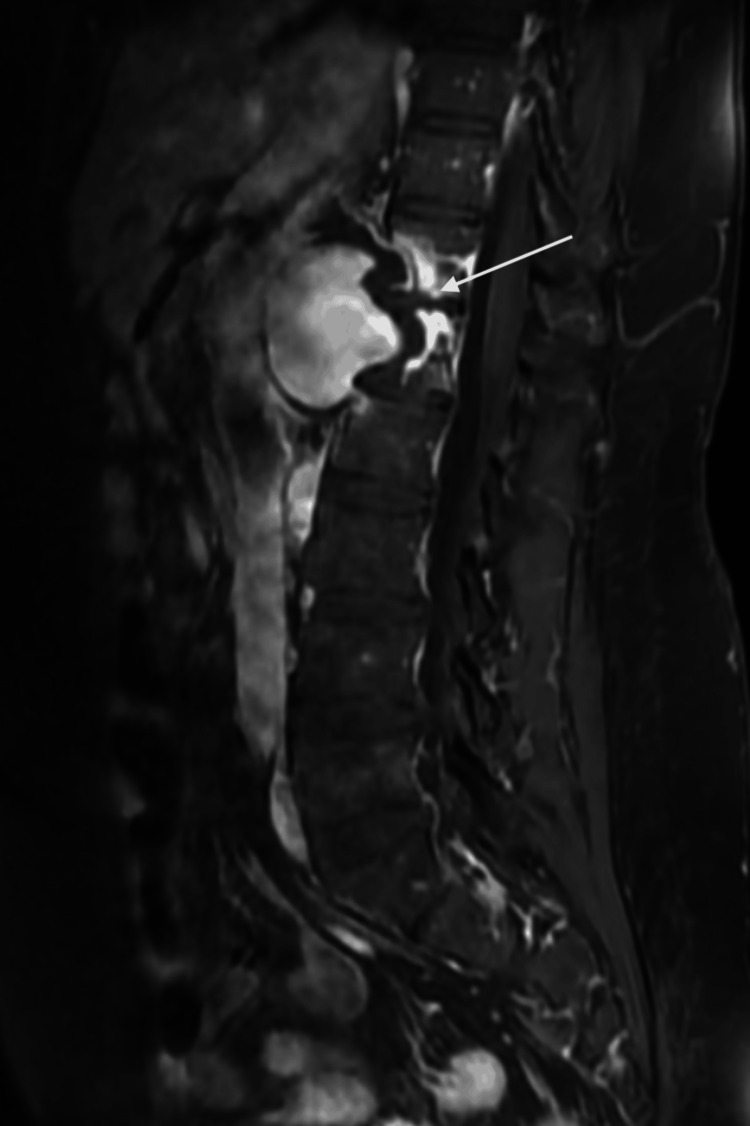
Sagittal post-gadolinium T1-weighted MRI of the thoracolumbar spine The contrast-enhanced sequence shows intense heterogeneous enhancement of the affected D12-L1 disk and adjacent vertebral bodies, confirming the active inflammatory and infectious nature of the spondylodiscitis. It delineates the anterior soft-tissue infectious extension breaching the aortic wall to form a prominent, lobulated mycotic pseudoaneurysm (white arrow) MRI: magnetic resonance imaging

**Figure 3:**
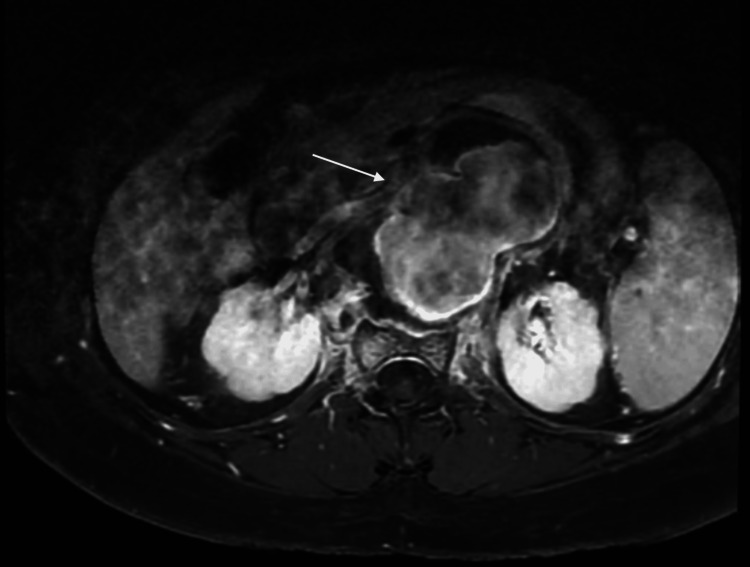
Axial post-gadolinium fat-saturated T1-weighted MRI at the D12-L1 level The axial view provides a clear visualization of the irregularly shaped saccular outpouching arising from the anterior wall of the infrarenal abdominal aorta, measuring approximately 7.7 × 5 cm in maximum diameter. The image highlights the peripheral enhancement of the mycotic pseudoaneurysm and its intimate, destructive contact with the prevertebral space (white arrow) MRI: magnetic resonance imaging

Emergency vascular surgery consultation was obtained, and the patient was admitted to the ICU. The plan was for urgent endovascular aortic repair as a bridge to definitive open surgical debridement. Empirical broad-spectrum antibiotic therapy was initiated with intravenous vancomycin (15 mg/kg load) and piperacillin-tazobactam (4.5 g q6h) pending susceptibility results. However, this antibiotic regimen was administered for only a few hours due to the patient's rapid demise. Over the ensuing four hours, the patient's hemodynamic status deteriorated precipitously despite aggressive fluid resuscitation and vasopressor support with norepinephrine and vasopressin. She developed refractory septic shock with multiorgan dysfunction - including acute kidney injury, lactic acidosis (serum lactate 12 mmol/L), and respiratory failure requiring emergent endotracheal intubation. Given the degree of hemodynamic instability, the surgical team judged the operative risk to be prohibitive. The patient died within eight hours of initial diagnosis. Permission for autopsy was declined by the family.

## Discussion

Aortic involvement complicating vertebral osteomyelitis/spondylodiscitis is an exceptionally rare and uniformly devastating event. To our knowledge, fewer than 100 cases of vertebra-aortic fistula or contiguous mycotic aortic pseudoaneurysm secondary to spinal infection have been described in the literature in the English language, the vast majority being reported as isolated case reports [5-8]. Our case adds to this sparse but critically important body of literature.

The pathogenesis of aortic involvement in spondylodiscitis follows one of two principal mechanisms. First, direct contiguous spread may occur when the pyogenic inflammatory process - driven by proteolytic enzymes, cytokine-mediated tissue destruction, and bacterial virulence factors - erodes through the anterior longitudinal ligament and the prevertebral fascia into the adventitia of the adjacent aorta, ultimately breaching the full thickness of the aortic wall [5,9]. Second, bacteremic seeding of a pre-existing aortic atherosclerotic plaque or aneurysm may create a mycotic pseudoaneurysm that subsequently involves the adjacent vertebral column in a retrograde fashion [9,10]. The anatomical proximity of the thoracolumbar vertebral bodies to the abdominal aorta - particularly at the L1-L4 levels, where the aorta is in direct contact with the prevertebral fascia - explains why the infrarenal aorta is the most commonly affected segment in such cases [6].

*S. aureus*, as in our patient, is the predominant causative organism in reported cases of aortic involvement complicating spondylodiscitis [7]. Its pathogenicity is largely attributable to its broad array of virulence factors, including surface adhesins (fibronectin-binding proteins and collagen adhesin) that facilitate tissue invasion, as well as alpha-toxin and leucocidins that directly disrupt host cell membranes and vascular endothelium [11]. The combination of these attributes renders *S. aureus *particularly adept at producing rapid, destructive tissue necrosis - a prerequisite for fistula formation.

From a diagnostic standpoint, contrast-enhanced MRI of the spine remains the modality of choice for the evaluation of suspected spondylodiscitis, offering superior soft-tissue contrast resolution and multiplanar capability [12]. In the setting of known or suspected vascular involvement, cross-sectional vascular imaging - specifically CT angiography (CTA) or contrast-enhanced MR angiography - is essential for delineating the extent of aortic wall involvement, characterizing the morphology of any associated pseudoaneurysm, and planning endovascular or surgical intervention [13]. In our patient, the MRI incidentally but definitively demonstrated the vertebra-aortic fistula and mycotic pseudoaneurysm, which had not been suspected clinically. This underscores the importance of meticulous review of the prevertebral soft tissues and adjacent vascular structures when interpreting spinal MRI in the context of spondylodiscitis.

Management of this combined pathology is extraordinarily challenging and requires seamless coordination between infectious disease specialists, spine surgeons, vascular surgeons, and intensivists. Contemporary case series and systematic reviews suggest that combined endovascular or open aortic repair with surgical debridement of the infected spinal segment, followed by prolonged pathogen-directed antibiotic therapy (typically 6-12 weeks), affords the best chance of survival [5,8,14]. Endovascular approaches - particularly thoracic or abdominal endovascular aortic repair (TEVAR/EVAR) - have been advocated as a temporizing measure in hemodynamically unstable patients, providing immediate hemorrhagic control as a bridge to definitive open repair and spinal debridement, though long-term outcomes with endovascular stent grafts in an infected field remain suboptimal owing to the risk of persistent infection and device-related complications [14]. Despite optimal multidisciplinary management, reported overall mortality in this subset of patients exceeds 50-80%, with the highest mortality observed in those presenting in frank septic shock, as illustrated by our case [5,7,8].

Several important clinical lessons emerge from this case. First, the possibility of great vessel involvement should be actively considered in any patient with spondylodiscitis who presents with unexplained hemodynamic instability, rapidly progressive systemic sepsis, or an abdominal mass, even in the absence of overt hemorrhage, as the pseudoaneurysm may remain intact until the terminal stages. Second, prompt cross-sectional vascular imaging should be obtained whenever spinal MRI raises suspicion of anterior soft-tissue extension toward the aorta. Third, definitive multidisciplinary intervention - ideally including endovascular aortic repair and intensive antibiotic therapy - must be initiated without delay, as the window for successful intervention is exceedingly narrow. Finally, this case re-emphasizes that spondylodiscitis, while often perceived as a subacute or chronic illness, can pursue a fulminant course with lethal vascular complications.

## Conclusions

We presented a fatal case of *S. aureus* spondylodiscitis at the T12-L1 level complicated by direct fistulization into the abdominal aorta and the formation of a mycotic pseudoaneurysm, resulting in refractory septic shock and death despite immediate multidisciplinary involvement. Vertebro-aortic fistula and mycotic pseudoaneurysm represent some of the rarest and most catastrophic complications of spinal infection. A high index of clinical suspicion, prompt cross-sectional vascular imaging, and urgent combined vascular and spinal surgical intervention, ideally instituted before the onset of hemodynamic compromise, are critical factors that may improve the chances of survival.
